# APOE-ε4 moderates the association between diet quality and executive function in middle-aged women at increased risk for Alzheimer’s disease

**DOI:** 10.1017/jns.2026.10106

**Published:** 2026-06-08

**Authors:** Chadsley M. Wessinger, Kyoung Shin Park, Samantha L. DuBois, Laurie Wideman, Brittany D. Armstrong, Jarod C. Vance, Samuel W. Kibildis, Hadassah Som-Pimpong, Jennifer L. Etnier

**Affiliations:** 1 Kinesiology, The University of North Carolina at Greensboro, USA; 2 Emory University School of Medicine, USA; 3 Kinesiology, Appalachian State University, USA; 4 Health, Recreation, and Kinesiology, Longwood University, USA; 5 Athens-Clarke County Unified Government, USA; 6 Meharry Medical College School of Medicine, USA

**Keywords:** Apolipoprotein E, Cognition, Dementia, Dietary patterns, Fatty acids, Vegetables

## Abstract

We examined whether diet quality is associated with executive function (EF) in middle-aged women with a family history of Alzheimer’s disease (AD) and whether this is moderated by a genetic AD risk factor, APOE-ε4 allele carriage. Data from 102 low-physically active women (*Age_Mean_* = 56.9 ± 5.8 years) were analyzed cross-sectionally. Diet quality was quantified via the Healthy Eating Index-2020 (HEI-2020). Overall EF and sub-domains (working memory [WM], exogenous inhibitory control [ICex], endogenous inhibitory control [ICen], and cognitive flexibility) were measured using neurocognitive assessments. APOE-ε4 carriage was determined from saliva. Moderated regression models tested HEI-2020*APOE-ε4 interactions; significant (*p* < .05) and near-significant (*p* < .10) interactions were followed by simple slope analyses. Main effects of HEI-2020 predicted overall EF (*p* = .033) and WM (*p* = .003). Interaction effects of HEI-2020*APOE-ε4 for EF and WM (*p’s* = .056–.038) were such that diet quality was positively associated with overall EF (*p* = .003) and WM (*p* = .005) in noncarriers only. Main effects of select HEI-2020 sub-scores were observed for overall EF and sub-domains (*p’s* = .001–.047). Interaction effects of total vegetables*APOE-ε4 on overall EF and ICex, saturated fatty acids*APOE-ε4 on overall EF and WM, and added sugars*APOE-ε4 on ICen (*p’s* = .007–.074) were such that sub-scores were positively associated with cognition in noncarriers only (*p’s* < .05). Diet quality and select sub-scores were positively associated with EF in middle-aged women without APOE-ε4 only. Future work should explore if APOE-ε4 carriers require a threshold of diet quality to yield cognitive benefits.

## Introduction

The recent Lancet Standing Commission on dementia prevention identified cardiovascular and metabolic risk factors (e.g. cardiovascular disease, diabetes) as key risk factors for Alzheimer’s disease (AD).^(1)^ Importantly, these risk factors are strongly influenced by dietary intake. According to the Dietary Guidelines for Americans (DGA), high-quality dietary patterns include higher intake of fruits, vegetables, whole grains, lean proteins, sources of omega-3 fatty acids (FA), and limited intake of sources of saturated FA and processed foods to mitigate the risk for chronic disease.^(2)^ The Healthy Eating Index (HEI) is a widely used tool to assess how well a diet aligns with these recommendations.^(3)^ While the definitions of diet quality vary, studies have commonly used composite indices like the HEI or examined specific components (e.g. vegetable, fruit, saturated FA intake) to assess their distinctive impacts on cognitive health. Longitudinal cohort studies in midlife and older ages have shown that high diet quality is protective against cognitive impairment.^(4,5)^

The cognitive domain executive function (EF) refers to top-down cognitive processes essential for overall health and well-being that support goal-oriented behaviour.^(6)^ EF can be further stratified into the sub-domains of working memory (WM), inhibitory control, and cognitive flexibility. WM refers to the temporary storage, manipulation and retrieval of information.^(7)^ Inhibitory control can be further stratified into exogenous inhibitory control (i.e. the ability to automatically suppress attention to distracting, stimulus-driven inputs) and endogenous inhibitory control (i.e. the ability to voluntarily suppress attention or responses based on internal goals or task demands).^(6)^ Cognitive flexibility refers to the ability to shift between tasks or sets of information.^(6)^ Importantly, EF decline has been implicated as a prodromal marker of AD,^(8)^ thus, investigating how diet quality relates to EF sub-domains may provide insight into potential early, modifiable pathways through which lifestyle behaviours may impact cognitive function and thereby risk for AD.

Cross-sectional associations between diet quality and cognition across various stages of adulthood have been observed with evidence that high diet quality is associated with better cognitive performance across the domains of EF and memory in middle-aged and older adults.^(9,10)^ Further, adherence to the Mediterranean-Dietary Approaches to Stop Hypertension Intervention for Neurogenerative Delay is associated with faster information processing, among middle-aged adults.^(11)^ Also, decrements to information processing often coincide with decrements to EF.^(12)^ Population-level studies, including samples representative of the U.S. and Hispanic/Latino-focused samples, have shown that better HEI scores are associated with better global cognition, EF, and memory performance.^(9,10,13)^ The importance of high diet quality in midlife is of particular interest given its association with a lower risk of cognitive decline in later life.

Along with lifestyle factors, genetic variants play a critical role in assessing the risk for AD. Inheriting at least one copy of the apolipoprotein E epsilon 4 allele (APOE-ε4) increases the risk of developing AD by two to three times.^(14–16)^ APOE-ε4 carriage is also associated with cognitive deficits in older ages in preclinical/prodromal stages due to early AD pathologies.^(17,18)^ Importantly, it has been suggested that subtle cognitive changes in APOE-ε4 carriers can occur in midlife, prior to the onset of the prodromal period of AD.^(19–21)^ Studies conducted by our research team^(22)^ and others^(23)^ have demonstrated that APOE-ε4 carriage is associated with poorer cognitive performance in cognitively unimpaired, middle-aged adults. Therefore, midlife may be a critical window when genetically induced, subtle cognitive changes may occur, thus representing a crucial age range to investigate how lifestyle factors, such as diet quality, can play a role in buffering these early changes.

To date, studies on the interplay between diet quality, APOE-ε4 carriage, and cognition have provided heterogeneous evidence. Some studies of older adults, like a cross-sectional study of older adults (69.4 ± 3.8 years, 61.5% female) by Felisatti et al.^(24)^, have reported positive relationships between diet quality and attention – but not memory or EF – only for APOE-ε4 carriers. In contrast, others have reported positive relationships for both carriers and noncarriers including a longitudinal cohort study of older adults linking diet quality with EF (69.3 ± 6.4 years, 60.2% female)^(25)^ and a high-diet quality intervention study in older adults also demonstrating benefits for EF (67.0 ± 6.0 years, 55.5% female).^(26)^ In a population-based longitudinal cohort study (*N* = 1,449; mean age at baseline: 50.6 ± 6.0; mean age at follow-up: 71.6 ± 4.1), Kivipelto et al.^(27)^ observed that APOE-ε4 carriers with poor diet quality during midlife – characterized by low intake of polyunsaturated FA and high intake of saturated FA – had increased odds of developing dementia (low polyunsaturated FA intake: OR = 1.95[0.37–10.40]; high saturated FA: OR = 11.29[0.82–155.94]) compared to the referent groups of carriers with high intake of polyunsaturated FA and carriers with low intake of saturated FA, respectively. No significant associations were observed among APOE-ε4 noncarriers, suggesting that midlife dietary intake may more strongly influence long-term cognitive outcomes later in life in APOE-ε4 carriers compared to noncarriers. Given that prior research has primarily reported cognitive outcomes in older adults, it is unclear if the early deleterious effects of APOE-ε4 on cognition at midlife can be offset by higher diet quality.

Given that a greater proportion of women are affected by AD compared to men^(28,29)^ and that a family history of AD (FH+) further increases the risk of AD,^(30,31)^ middle-aged women with FH+ are an important at-risk population in which to investigate the extent to which diet quality is associated with early variation in cognitive performance as a function of APOE-ε4 carriage. This study aimed (1) to determine the cross-sectional association between diet quality and EF in middle-aged women with FH+; and (2) to investigate the extent to which the diet-EF association was moderated by APOE-ε4 carriage. We hypothesized that diet quality would be positively associated with EF in this sample. Further, although some population-based, prospective studies report diet quality-related cognitive benefits regardless of APOE-ε4 carriage,^(32,33)^ other work suggests the diet quality-cognition relationship may be stronger in APOE-ε4 carriers.^(27,34)^ Therefore, we expected positive associations between diet quality and cognitive performance among APOE-ε4 carriers, with these associations attenuated or absent among noncarriers.

## Methods

### Study design and participants

**Figure 1.**
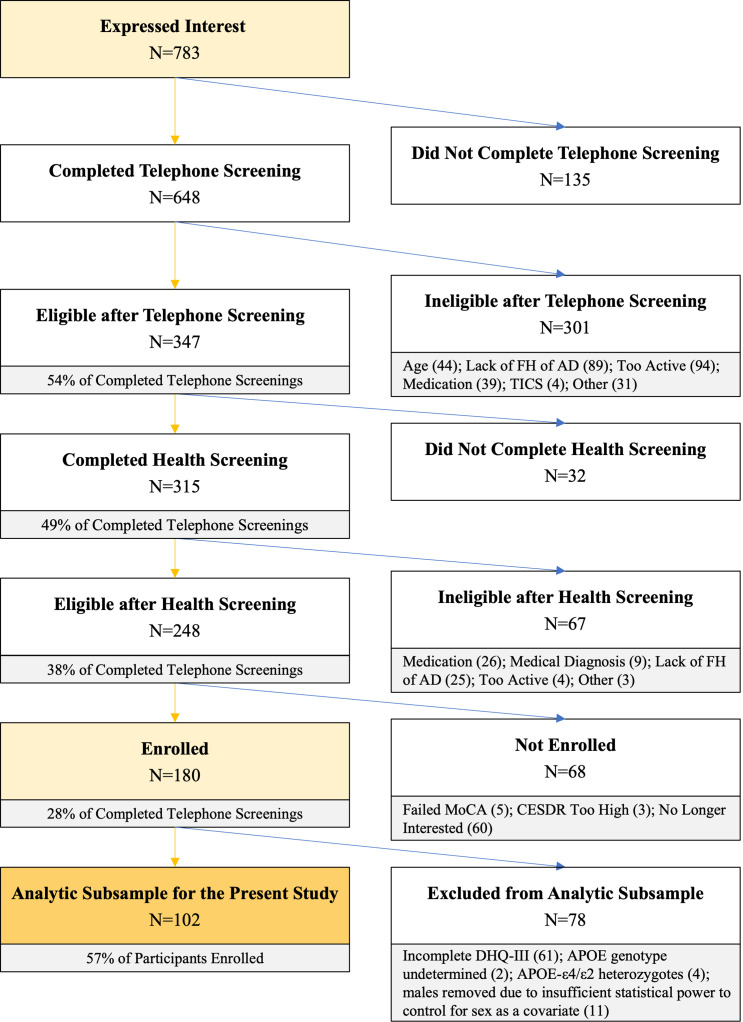
CONSORT-style participant flow diagram.

We analysed the baseline data from 102 middle-aged women (40–65 years old) enrolled in a clinical trial (NCT03876314). See Figure 1 for a CONSORT-style diagram outlining the initial sample (*n* = 180) and analytic sample (*n* = 102). This subset was comprised of primarily white (91.2%), non-Hispanic (96.1%), college educated (16.3 ± 2.0 years of education), middle-aged (56.9 ± 5.8 years) women. Participants self-reported at least one first-degree relative or two second-degree relatives with FH+, were cognitively unimpaired, and were low-physically active (<90 min/week moderate-to-vigorous physical activity). Cognitive status was assessed during telephone screening using the Modified Telephone Interview for Cognitive Status (TICS-m) with a cutoff point of 33^(35)^ and, following the provision of informed consent, via Montreal Cognitive Assessment (MoCA) with a cutoff point of 26.^(36)^ Participants who scored above both TICS-m and MoCA cutoffs and did not self-report any history of cognitive impairment were classified as cognitively unimpaired. Detailed inclusion and exclusion criteria were described previously.^(37)^ At baseline, participants completed lab visits for APOE-ε4 genotyping and cognitive testing with demographics and health history data collected prior to the visits. Dietary intake was assessed via a food frequency questionnaire post lab visit. The study was conducted according to the guidelines laid down in the Declaration of Helsinki and all procedures were approved by the University of North Carolina at Greensboro Institutional Review Board (IRB number: 18-0228). All participants provided written informed consent prior to participation. We used the STROBE-nut reporting guidelines.^(38)^

### Predictors

#### Genetic sampling

APOE genotype was determined from passive drool saliva samples collected using Orangene Discover salivary collection kits (ORG-500; DNA Genotek, Ontario, Canada). To prevent enzymatic degeneration of deoxyribonucleic acid (DNA) degradation, samples were immediately mixed with the provided additive. Samples were sent to the Wake Forest School of Medicine, Genomics Core Lab where genomic DNA was extracted for analysis of two single nucleotide polymorphisms (SNPs), codons 112 and 158, used to classify participants as carriers or noncarriers of an APOE-ε4 allele. Due to potential confounding effects of APOE-ε2 allele carriage on AD risk,^(39)^ heterozygous APOE-ε4/ε2 carriers (*n* = 4) were removed from the subsample (see Figure 1). The analytic sample included 61 noncarriers and 41 carriers.

#### Diet quality

Following genetic sampling and cognitive testing, participants were contacted by email to complete a dietary assessment survey. Dietary intake data were collected and analysed using the Diet History Questionnaire III (DHQ-III), a web-based tool developed by the National Cancer Institute, Bethesda, MD (https://epi.grants.cancer.gov/dhq3). Using unique web-links, participants self-reported their food intake over the past month including estimated serving sizes. HEI-2020 scores were computed directly from the DHQ-III database.^(3)^

Because there is evidence to suggest that certain HEI sub-scores (e.g. fruit, FA intake) may be positively associated with cognitive performance,^(40)^ HEI-2020 sub-scores were also calculated. These sub-scores were classified relative to the DGA, recommending adequacy (total fruits, whole fruits, total vegetables, greens and beans, whole grains, dairy, total protein foods, seafood and plant proteins, ratio of unsaturated FA to saturated FA [FA ratio]) and moderation (refined grains, sodium, added sugars, saturated FA) components. For the adequacy components (AdComp), higher intake and a higher FA ratio are associated with better diet quality (i.e. consuming these components in adequate quantities); for the moderation components (ModComp), lower intake is indicative of better diet quality (i.e. consuming these components in moderate quantities). Scores for these components range from 0 to 5 or from 0 to 10 with higher scores indicating better diet quality for both AdComp and ModComp. These sub-scores are scored separately and summed, resulting in an overall HEI-2020 from 0 to 100. Additional scoring details are provided in Supplemental Table 1.

### Outcome measures

#### Cognitive testing

Participants underwent cognitive assessment following genetic sampling during the same or separate lab visits.^(37)^ The tests were administered via paper/pencil, desktop computer (Dell, OptiPlex GX110), and/or tablet computer (iPad 12.0, Apple Inc.). A detailed description of the specific cognitive tests and their respective protocols is available elsewhere.^(37)^ Because EF is among the cognitive domains most consistently linked to diet quality, the primary cognitive outcomes of this study were measures of EF (i.e. WM, inhibitory control, and cognitive flexibility). WM was assessed via NIH-Toolbox List Sort Working Memory Test (LSWMT),^(41)^ Digit Span-Backwards (DGB),^(42)^ and the IGNITE Spatial Working Memory Test (SWM).^(43)^ Exogenous inhibitory control (ICex) was assessed via NIH-Toolbox Flanker Test (Flanker)^(44)^ and the IGNITE version of the Stroop Colour-Word Test (Stroop).^(45)^ Endogenous inhibitory control (ICen) was assessed via Symbol Digit Modalities Test (SDMT)^(46)^ and the Paced Auditory Serial Addition Test (PASAT).^(47)^ Cognitive flexibility was assessed using the Dimensional Change Card Sort task^(44)^ and the Trail Making Task B (TMT-B).^(48)^ Scores on each cognitive assessment were converted to *z*-scores and averaged to produce composite scores of WM, ICex, ICen, cognitive flexibility, and an overall EF score. The overall EF score was derived by averaging the *z*-scores across all cognitive assessments. The model including overall EF was considered our primary analytic model because it provides a robust, global index of EF and reduces measurement error. However, because the sub-domains of EF represent distinct processes, additional analyses explored EF sub-domains individually. See Table 1 for a summary.

**Table 1.** Summary of cognitive assessments and outcome variables used to compute each composite score Table 1 long description.

Sub-domain of executive function	Cognitive task	Outcomes
Working memory	SWM	3-Item accuracy 4-Item accuracy
	LSWMT	Total correct
	DGB	Total correct
Exogenous inhibitory control	Flanker	Computed score (composite of accuracy and reaction time)
	Stroop	Incongruent response time^‡^
Endogenous inhibitory control	SDMT	Total correct
	PASAT	Total correct 2-sec condition
Cognitive flexibility	DCCS	Computed score (composite of accuracy and reaction time)
	TMT-B	Time to trail completion^‡^

SWM, IGNITE Spatial Working Memory Test; LSWMT, NIH-Toolbox List Sort Working Memory Test; DGB, Digit Span Backwards Test, SDMT, Symbol Digit Modalities Test; PASAT, Paced Auditory Serial Addition Test; DCCS, NIH-Toolbox Dimensional Change Card Sort; TMT-B, Trail Making Task B.

‡ Stroop incongruent response time and TMT-B trail completion time variables were reversed so that higher values reflected faster times, interpreted as better performance.

### Statistical analyses

To compare participant characteristics between APOE-ε4 carriers and noncarriers, independent samples *T*-tests were conducted for quantitative variables (age, years of education, MoCA, BMI, HEI-2020 and its sub-scores, and cognitive composite scores), and chi-square tests for categorical variables (race, ethnicity).

Using R (v2025.05.0, Posit Software, PBC), data were first visualized to assess distributions and identify potential outliers. All outliers at ≥3 standard deviations were winsorized using the winsorZ function. Simple moderated regression models were fitted using the PROCESS macro for R^(49)^ to test the main effects of mean-centred HEI-2020 and APOE-ε4 carriage and their interaction on EF. Independent models were run for each composite score (see Table 1), with age and education included as covariates. While it is not clear if BMI consistently improves model fit when assessing the relationship between diet quality and cognitive performance,^(10,25)^ it was also considered for use as a covariate in the models. Moderation models exhibiting statistically significant (*p* < .05) results were then examined for significant or near-significant (*p* < .10) interaction effects between HEI-2020 and APOE-ε4 carriage. When interaction effects met these thresholds, they were explored further using simple slope analyses to probe conditional effects of HEI-2020 based on APOE-ε4 carriage. Effect sizes were used as an additional indicator of practical significance to aid in interpreting significant and near-significant results and to inform future replication efforts. While we tested hypotheses regarding both main and interaction effects, given that main effects in moderation models are conditional on the moderator, and can therefore be misleading to interpret in isolation, we report effect sizes only for the interaction terms of HEI-2020*APOE-ε4 carriage using the formula, $f^{2}=\frac{\Delta R^{2}}{\left(1-R^{2}\right)}$ . Effect sizes were interpreted as .02 (small), .15 (medium), and .35 (large).^(50)^

**Figure 2.**
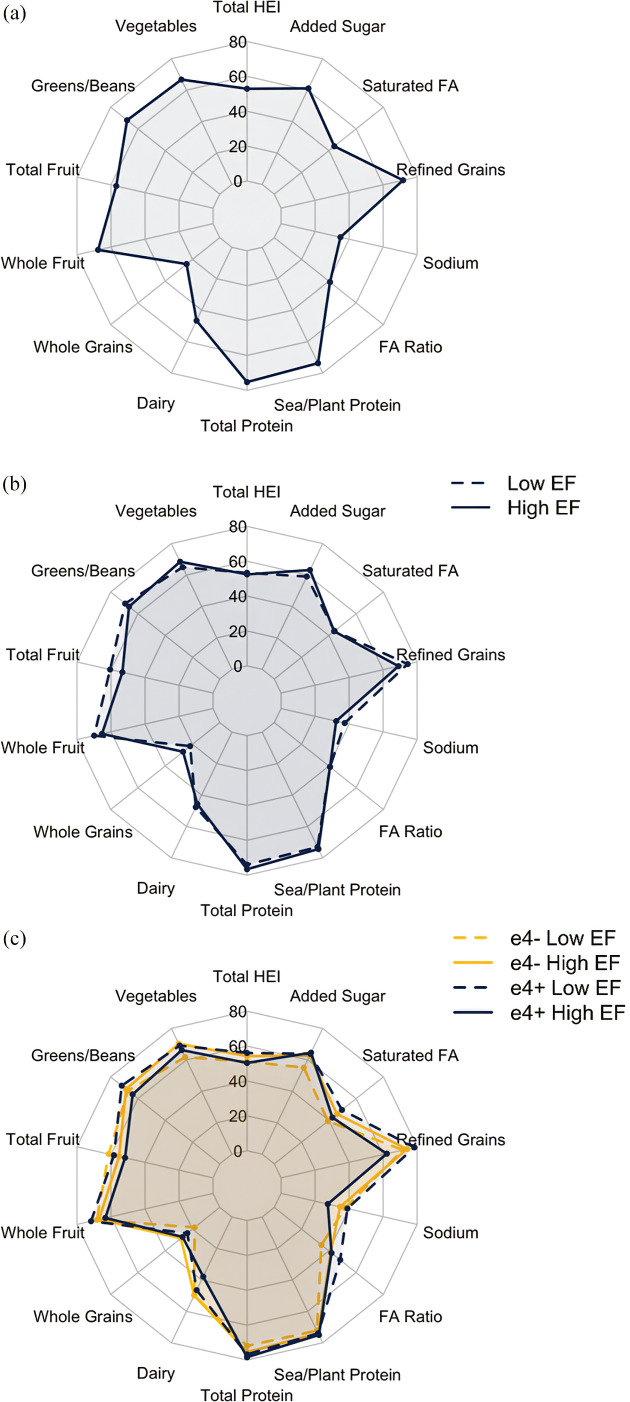
HEI-2020 radar plots across (a) the total sample, (b) low vs high executive function performers, and (c) low vs high executive function performers stratified by APOE-ε4 carriage. EF, composite executive function scores; e4-, APOE-ε4 noncarriers; e4+, APOE-ε4 carriers; HEI, healthy eating index-2020; FA, fatty acids.

HEI-2020 overall and sub-scores are displayed for the full sample, stratified by overall EF performance median split, and APOE-ε4 carriage in Figure 2a–c, respectively. Five sub-scores were selected for regression analyses based on visual inspection of radar plots grouped by APOE-ε4 carriage and median split across overall EF composite score. Because there are 13 HEI-2020 sub-scores, in an effort to limit the number of comparisons, selection was guided by previous literature,^(10,27,51)^ apparent divergence between low and high cognitive groups in ε4-stratified plots, indicating potential cognitive sensitivity to specific dietary components represented by specific HEI-2020 sub-scores, and only sub-scores without between group differences by APOE-ε4 carriage were eligible for selection. Radar plots for the EF sub-domains are displayed in Supplemental Figures 1–2. The five sub-scores selected were adequacy of vegetable intake, total fruit intake, and FA ratio, and moderation of saturated FA intake and added sugars intake. The sub-scores were dichotomized given their non-normal, negatively skewed distribution. For total fruit and total vegetables, the sub-scores were split by whether participants met the DGA (i.e. score of 5.0) or not. Participants were classified as meeting (*n* = 41) vs. not meeting (*n* = 61) the DGA for total fruit and as meeting (*n* = 43) vs. not meeting (*n* = 59) the DGA for total vegetables. The remaining three sub-scores were dichotomized via median split due to imbalanced group sizes when using DGA cutoffs. Dichotomized sub-scores were included as predictors for regression models as described for the overall HEI-2020 score.

## Results

### Participant characteristics

Sample characteristics stratified by APOE-ε4 carriage are provided in Table 2. There were no between group differences across demographic, HEI-2020, and composite EF scores. The dietary pattern of the sample is illustrated in Figure 2a. The total HEI-2020 of 66.0 ± 10.7 for this sample aged 40–65 years is higher than population-level data of adults (57.0 in those between ages 19 and 59 years) from NHANES 2017–2018. However, in the same dataset, the average HEI-2020 score for adults aged 60+ years was 61.0,^(3)^ aligning more closely with our sample. The mean HEI-2020 sub-scores are provided for APOE-ε4 carriers and noncarriers in Supplemental Table 2. APOE-ε4 noncarriers had higher dairy scores compared to carriers (*p* = .046), and there was a near significant trend for higher total protein scores in carriers compared to noncarriers (*p* = .062). There were no differences in the other sub-scores (*p’s* > .151).

**Table 2.** Participant characteristics Table 2 long description.

Variable	APOE-ε4 Carriers (*n* = 41)		APOE-ε4 Noncarriers (*n* = 61)		Significance
	Mean	SD	Mean	SD	
Age, years	56.7	5.9	57.0	5.8	.791
MoCA	28.2	1.2	28.1	1.6	.709
Education, years	16.4	1.9	16.2	2.0	.686
BMI, kg/m^2^	28.7	5.5	27.8	5.2	.393
Race	–		–		.688
White					
*n*	37		56		–
%	91.2		91.8		–
Black					
*n*	4		3		–
%	9.8		4.9		–
Multiracial					
*n*	0		1		–
%	0		1.6		–
Not Reported					
*n*	0		1		–
%	0		1.6		–
Ethnicity	–		–		.534
Hispanic					
*n*	1		2		–
%	2.0		3.3		–
Not Hispanic or Latino					
*n*	40		58		–
%	98.0		95.1		–
Not Reported					
*n*	0		1		–
%	0		1.6		–
HEI-2020	66.4	9.2	65.7	11.7	.736
Executive function (Composite *z*-score)	0.06	0.64	−0.04	0.64	.431
Working memory (Composite *z*-score)	0.02	0.78	−0.02	0.68	.781
Exogenous inhibitory control (Composite *z*-score)	−0.02	0.74	0.01	0.83	.849
Endogenous inhibitory control (Composite *z*-score)	0.06	0.86	−0.04	0.90	.548
Cognitive flexibility (Composite *z*-score)	0.20	0.67	−0.14	0.84	.024^*^

APOE-ε4, apolipoprotein E epsilon 4 allele; MoCA, Montreal Cognitive Assessment; HEI-2020, Healthy Eating Index-2020.

Between group differences were tested via independent samples t-tests for quantitative variables and chi-squared difference tests for categorical variables.

* indicates statistically significant differences (*p* < .05).

### Overall HEI-2020 as the focal predictor

**Figure 3.**
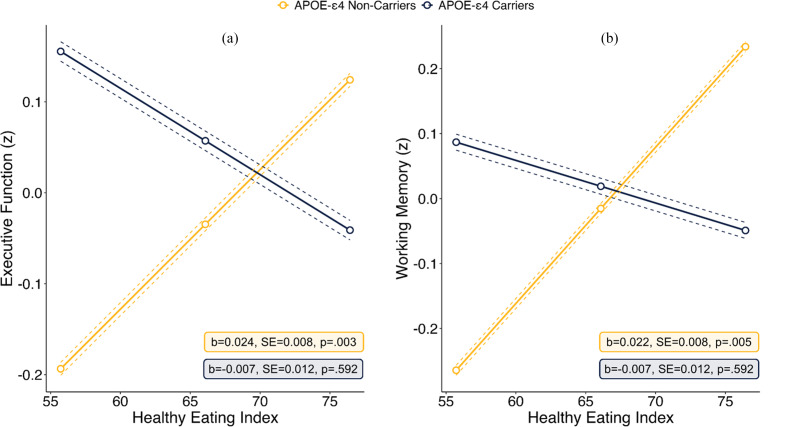
Conditional simple slope effects of HEI-2020 on (a) overall executive function and (b) working memory stratified by APOE-ε4 carriage. Among APOE-ε4 noncarriers, higher HEI-2020 scores predicted better executive function and working memory performance, while this association was not significant among carriers. Solid lines represent simple slopes, open points represent levels (−1 SD, mean, and + 1 SD) of HEI-2020 scores, and dotted lines represent the standard error of the slope. The slope (*b*), standard error, and significance (*p*) values for each line are overlaid in the bottom right of each plot.

The model predicting overall EF was significant (*p* = .019), with a significant main effect of HEI-2020 (*p* = .033), no main effect of APOE-ε4 carriage (*p* = .459), and a near-significant interaction of HEI-2020 and APOE-ε4 carriage (*p* = .056) yielding a small effect size (*f*^2^ = 0.039), corresponding with a significant change in explained variance (*ΔR*^2^ = 0.034). Age was negatively associated with EF performance (*p* = .010), whereas education was not significantly associated with EF (*p* = .829). Simple slope analysis revealed that HEI-2020 was positively associated with EF in APOE-ε4 noncarriers (*p* = .033) but not in APOE-ε4 carriers (*p* = .378). The simple slopes are plotted in Figure 3a, and a scatter plot of the raw data is shown in Supplemental Figure 3. The model predicting WM was significant (*p* = .045). There was a significant positive main effect of HEI-2020 (*p* = .003), no significant main effect of APOE-ε4 carriage (*p* = .828), and a significant interaction effect of HEI-2020 and APOE-ε4 carriage on WM (*p* = .038) with a small effect size (*f*^2^ = .046), corresponding with a significant change in explained variance of *ΔR*^2^ = 0.041. Neither age nor education had a significant effect on WM (*p’s* ≥.220). The significant interaction effect was followed up with simple slope analyses (Figure 3b and Supplemental Table 3), and a scatter plot of the raw data is shown in Supplemental Figure 4. The simple slope analyses revealed a positive relationship between HEI-2020 and WM in APOE-ε4 noncarriers (*p* = .003), while there was no significant relationship in APOE-ε4 carriers (*p* = .592). The model fits predicting ICex and cognitive flexibility were not significant (*p* = ≥ .111) whereas the model fit predicting ICen was significant (*p* = .002). For ICen, there was no significant main effect of HEI-2020 or APOE-ε4 carrier status (*p’s* ≥ .288), and there was no significant interaction effect of HEI-2020 and APOE-ε4 carriage (*p* = .168). There was a significant negative effect of age (*p* = .001) but no effect of education (*p* = .165).

**Figure 4.**
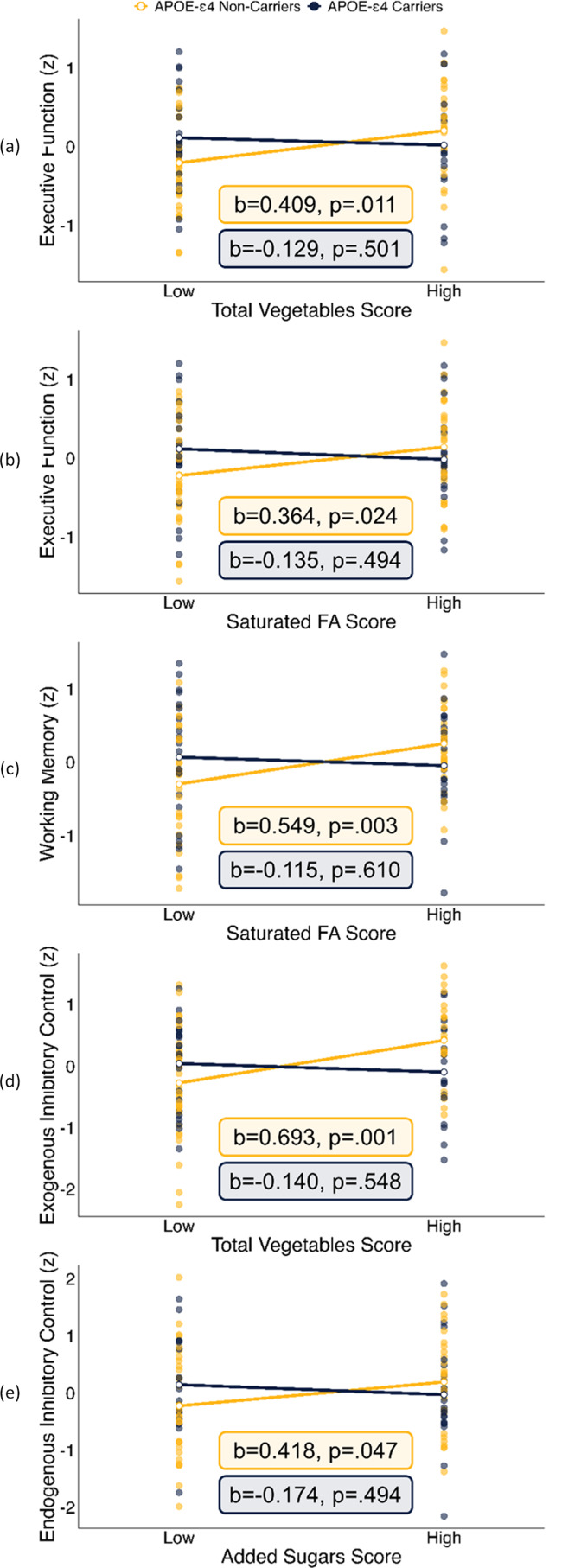
Conditional simple slope effects of HEI-2020 sub-scores stratified by APOE-ε4 carriage. Among APOE-ε4 noncarriers, (a) higher vegetable scores predicted overall executive function, (b) higher saturated FA scores predicted better overall executive function, (c) higher saturated FA scores predicted better WM performance, (d) higher vegetable scores predicted better ICex, and (e) higher added sugars scores predicted better ICen. No significant associations were observed among APOE-ε4 carriers. Solid points represent individual participant data, solid lines represent simple slopes, and open points represent levels (Low, high) of HEI-2020 sub-scores. The slope (*b*) and significance (*p*) values for each line are overlaid in the bottom centre of each plot.

**Table 3.** Effects of HEI-2020 overall scores and the other predictors on composite cognitive scores Table 3 long description.

Predictor	*b*	*t*	*p*	*CI (LL, UL)*
***Overall executive function*** *R^2^* = .130, *F*(5, 96) = 2.862, *p* = .019^*^				
HEI-2020	0.0153	2.1685	.0326^*^	0.0013, 0.0294
APOE-ε4 carriage	0.0918	0.7442	.4586	−0.1530, 0.3365
HEI-2020^*^APOE-ε4 carriage	−0.0248	−1.9372	.0557^†^	−0.0502, 0.0006
Age	−0.0286	−2.6366	.0098^*^	−0.0501, −0.0071
Education	0.0071	0.2164	.8291	−0.0577, 0.0718
***Working memory*** *R^2^* = .110, *F*(5, 96) = 2.370, *p* = .045^*^				
HEI-2020	0.0242	3.0016	.0034^*^	0.0082, 0.0403
APOE-ε4 carriage	0.0307	0.2180	.8279	−0.2488, 0.3102
HEI-2020^*^APOE-ε4 carriage	−0.0308	−2.1065	.0378^*^	−0.0599, −0.0018
Age	−0.0153	−1.2351	.2198	−0.0399, 0.0093
Education	0.0209	0.5603	.5766	−0.0531, 0.0948
***Exogenous inhibitory control*** *R^2^* = .088, *F*(5, 96) = 1.848, *p* = .111				
HEI-2020	0.0130	1.4475	.1510	−0.0048, 0.0308
APOE-ε4 carriage	−0.0324	−0.2066	.8368	−0.3432, 0.2785
HEI-2020^*^APOE-ε4 carriage	−0.0181	−1.1116	.2691	−0.0504, 0.0142
Age	−0.0336	−2.4400	.0165^*^	−0.0610, −0.0063
Education	−0.0576	−1.3908	.1675	−0.1398, 0.0246
***Endogenous inhibitory control*** *R^2^* = .182, *F*(5, 96) = 4.282, *p* = .002^*^				
HEI-2020	0.0101	1.0688	.2878	−0.0087, 0.0289
APOE-ε4 carriage	0.0826	0.5002	.6181	−0.2452, 0.4105
HEI-2020^*^APOE-ε4 carriage	−0.0238	−1.3882	.1683	−0.0579, 0.0102
Age	−0.0525	−3.6126	.0005^*^	−0.0813, −0.0236
Education	0.0611	1.3991	.1650	−0.0256, 0.1478
**Cognitive flexibility** *R^2^* = .097, *F*(5, 96) = 2.068, *p* = .0760^†^				
HEI-2020	0.0042	0.4672	.6414	−0.0136, 0.0219
APOE-ε4 carriage	0.3407	2.1877	.0311^*^	0.0316, 0.6499
HEI-2020^*^APOE-ε4 carriage	−0.0187	−1.1581	.2497	−0.0509, 0.0134
Age	−0.0260	−1.8950	.0611^†^	−0.0532, 0.0012
Education	−0.0054	−0.1321	.8952	−0.0872, 0.0763

HEI-2020, Healthy Eating Index-2020; APOE-ε4, apolipoprotein E epsilon 4 allele.

* Indicates statistically significant results (*p* < .05).

† Indicates trend towards significance (*p* < .10).

The model *R*^2^, *F*-statistics, and unstandardized coefficients are reported in Table 3. Separate analyses were carried out with BMI as an additional covariate, but its inclusion did not significantly change model fit (*R*^2^*)* values or statistical significance. BMI was not significantly associated with EF outcomes, nor was it significantly associated with HEI-2020 or education (Supplemental Tables 4–5). Model fit statistics and a detailed model summary for overall EF with BMI included are presented in Supplemental Table 6, illustrating the lack of statistical significance of BMI as a predictor in this sample; results from the remaining models were consistent and are not shown. This indicates that HEI-2020 was associated with EF outcomes (overall and WM) independent of education and BMI in this sample.

### HEI-2020 sub-scores as the focal predictors

The model fit predicting overall EF by total vegetables was significant (*R*^2^ = .144, *F*[5,96] = 3.222, *p* = .010) with a significant main effect of total vegetables (*p* = .011), a trend level positive association for APOE-ε4 carriage (*p* = .052), and a significant interaction of total vegetables and APOE-ε4 carriage (p=.032). This interaction exhibited a small effect size (*f*^2^ = .0494, *ΔR*^2^ = 0.042). Simple slope analysis revealed that this interaction was driven by a positive association of total vegetables with overall EF in APOE-ε4 noncarriers only (*p* = .011; carriers *p* = .501; Figure 4a). The models for total fruit (*R*^2^ = .090, *F*[5,96] = 1.903, *p* = .101) and FA ratio (*R*^2^ = .104, *F*[5,96] = 2.222, *p* = .058) were not significant. The model for saturated FA (*R*^2^ = .130, *F*[5,96] = 2.877, *p* = .018) was significant. There was a significant main effect of saturated FA (*p* = .024) and trend level associations for a positive effect of APOE-ε4 carriage (*p* = .057) and for an interaction of saturated FA and APOE-ε4 carriage (*p* = .056), yielding a small interaction effect size (*f*^2^ = .0389, *ΔR*^2^ = 0.034). Simple slope analysis indicated that saturated FA was positively associated with overall EF in APOE-ε4 noncarriers (*p* = .024) but not APOE-ε4 carriers (*p* = .494); Figure 4b). The model for added sugars was significant (*R*^2^ = .121, *F*[5,96] = 2.637, *p* = .028), with a significant main effect of added sugars (*p* = .038) but no significant main effect of APOE-ε4 carriage (*p* = .147) or interaction of added sugars and APOE-ε4 carriage (*p* = .148).

The model fits predicting WM by total vegetables (*R*^2^ = .084, *F*[5,96] = 1.756, *p* = .130), total fruit (*R*^2^ = .042, *F*[5,96] = 0.840, *p* = .524), FA ratio (*R*^2^ = .065, *F*[5,96] = 1.338, *p* = .255), and added sugars (*R*^2^ = .029, *F*[5,96] = 0.567, *p* = .725) were not significant. However, the model predicting WM by saturated FA was significant (*R*^2^ = .109, *F*[5,96] = 2.360, *p* = .046) with a significant main effect of saturated FA (*p* = .003) and a trend towards a main effect of APOE-ε4 carriage (*p* = .074). Further, there was a significant interaction effect of saturated FA and APOE-ε4 carriage (*p* = .027) on WM with a small effect size (*f*^2^ = .053, *ΔR*^2^ = 0.047). Simple slope analyses revealed that there was a positive relationship between saturated FA and WM in APOE-ε4 noncarriers (*p* = .003), but not in carriers (*p* = .610; Figure 4c). There were no significant effects of age or education (*p’s* ≥ .113) in the models predicting WM.

The model predicting ICex by total vegetables was significant (*R*^2^ = .181, *F*[5,96] = 4.240, *p* = .002) with a significant main effect of total vegetables (*p* < .001), no main effect of APOE-ε4 carriage (*p* = .110), and a significant interaction effect of total vegetables and APOE-ε4 carriage (*p* = .007) with a small to medium effect size (*f*^2^ = .080, *ΔR*^2^ = 0.066). As illustrated in Figure 4d, simple slope analyses indicate that there was a positive relationship between total vegetable intake and ICex in APOE-ε4 noncarriers (*p* = .001), while there was no significant relationship in carriers (*p* = .548). The models including total fruit (*R*^2^ = .083, *F*[5,96] = 1.733, *p* = .134) and FA ratio (*R*^2^ = .084, *F*[5,96] = 1.768, *p* = .127) were not significant. The model for saturated FA was not significant (*R*^2^ = .094, *F*[5,96] = 1.986, *p* = .088). The model including added sugars was significant (*R*^2^ = .143, *F*[5,96] = 3.199, *p* = .010) and while there was a significant positive main effect for added sugars (*p* = .005), there was no main effect of APOE-ε4 carriage (*p* = .484) or interaction effect (*p* = .162). For all models predicting ICex, there was a significant negative effect of age (*p’s* = .008–.019), indicating that increased age was associated with worse cognitive performance, while there was no significant effect of education (*p’s* ≥ .073).

The model fits predicting ICen by total vegetables was significant (*R*^2^ = .185, *F*[5,96] = 4.349, *p* = .001) but there was no main effect of total vegetables (*p* = .308) or APOE-ε4 carriage (*p* = .174), and no significant interaction of total vegetables and APOE-ε4 carriage (*p* = .130). The model including total fruit was significant (*R*^2^ = .207, *F*[5,96] = 5.019, *p* < .001) with a significant negative main effect for total fruit (*p* = .027), no main effect of APOE-ε4 carriage (*p* = .406), and no interaction between total fruit and APOE-ε4 carriage (*p =* .139). The models for FA ratio (*R*^2^ = .189, *F*[5,96] = 4.480, *p* = .001) and saturated FA were significant (*R*^2^ = .178, *F*[5,96] = 4.149, *p* = .002), but there were no significant main effects or interactions (*p’s* ≥ .104). Finally, the model including added sugars was significant (*R*^2^ = .202, *F*[5,96] = 4.871, *p* < .001) with a positive main effect of added sugars (*p* = .047), no main effect of APOE-ε4 carriage (*p* = .120), and a near-significant interaction of added sugars by APOE-ε4 carriage (*p* = .074) with a small effect size (*f*^2^ = .034, *ΔR*^2^ = 0.027). Simple slope analyses revealed that there was a positive relationship between added sugars scores and ICen scores in APOE-ε4 noncarriers (*p* = .047) with no significant relationship in carriers (*p* = .494; Figure 4e). There was a significant negative main effect for age (*p’*s < .001) across all models predicting ICen with no significant effect of education (*p’*s ≥ .133).

The model fits predicting cognitive flexibility by total vegetables (*R*^2^ = .096, *F*[5,96] = 2.047, *p* = .079), total fruit (*R*^2^ = .0104, *F*[5,96] = 2.230, *p* = .057), and FA ratio (*R*^2^ = .084, *F*[5,96] = 1.767, *p* = .127) were not significant, whereas the models for saturated FA (*R*^2^ = .109, *F*[5,96] = 2.347, *p* = .047) and added sugars (*R*^2^ = .124, *F*[5,96] = 2.723, *p* = .024) were significant. For the model including saturated FA, there was no significant main effect of saturated FA (*p* = .351), there was a significant positive effect of APOE-ε4 carriage (*p* = .009), and no interaction of saturated FA and APOE-ε4 carriage (*p* = .107). For the added sugars model, there was a significant positive main effect of added sugars (*p* = .040) and APOE-ε4 carriage (*p* = .014) but no interaction of added sugars and APOE-ε4 carriage (*p* = .134). There was a significant negative main effect of age in the saturated FA and added sugars models (*p’s* = .027, .033) but no main effect of education (*p’s* ≥ .638).

Detailed results for the HEI-2020 sub-scores are shown in Table 4 and Supplemental Table 3. Separate analyses including BMI as a covariate were conducted; however, similar to models including overall HEI-2020, its inclusion did not change model fit or alter statistical significance for the HEI-2020 sub-score models. Therefore, these models are not presented.

**Table 4.** Effects of HEI-2020 sub-scores and the other predictors on composite cognitive scores Table 4 long description.

Predictor	*b*	*t*	*p*	*CI (LL, UL)*
***Overall executive function***				
Total vegetables	0.4091	2.5821	.0113^*^	0.0946, 0.7236
APOE-ε4 carriage	0.3181	1.9680	.0520^†^	−0.0028, 0.6389
Total vegetables^*^APOE-ε4 carriage	−0.5383	−2.1777	.0319^*^	−1.0290, −0.0476
Age	−0.0286	−2.6574	.0092^*^	−0.0499, −0.0072
Education	−0.0032	−0.1019	.9190	−0.0663, 0.0599
Total Fruit	−0.1137	−0.7115	.4785	−0.4309, 0.2035
APOE-ε4 carriage	−0.0075	−0.0470	.9626	−0.3246, 0.3096
Total Fruit^*^APOE-ε4 carriage	0.2888	1.0345	.3035	−0.2653, 0.8430
Age	−0.0296	−2.6831	.0086^*^	−0.0515, −0.0077
Education	−0.0010	−0.0303	.9759	−0.0663, 0.0643
FA ratio	0.2363	1.4649	.1462	−0.0839, 0.5565
APOE-ε4 carriage	0.1242	0.6730	.5026	−0.2421, 0.4905
FA ratio^*^APOE-ε4 carriage	−0.1130	−0.4407	.6604	−0.6221, 0.3961
Age	−0.0287	−2.6115	.0105^*^	−0.0505, −0.0069
Education	0.0084	0.2546	.7996	−0.0568, 0.0736
Saturated FA	0.3640	2.2869	.0244^*^	0.0481, 0.6799
APOE-ε4 carriage	0.3398	1.9258	.0571^†^	−0.0104, 0.6900
Saturated FA^*^APOE-ε4 carriage	−0.4986	−1.9329	.0562^†^	−1.0105, 0.0134
Age	−0.0326	−2.9844	.0036^*^	−0.0543, −0.0109
Education	0.0170	0.5190	.6049	−0.0479, 0.0819
Added sugars	0.3327	2.1068	.0377^*^	0.0192, 0.6461
APOE-ε4 carriage	0.2628	1.4617	.1471	−0.0941, 0.6198
Added sugars^*^APOE-ε4 carriage	−0.3639	−1.4581	.1481	−0.8593, 0.1315
Age	−0.0309	−2.8428	.0055^*^	−0.0525, −0.0093
Education	−0.0013	−0.0396	.9685	−0.0651, 0.0626
***Working memory***				
Total vegetables	0.4645	2.5098	.0138^*^	0.1208, 0.8120
APOE-ε4 carriage	0.2335	1.2370	.2191	−0.1350, 0.5960
Total vegetables^*^APOE-ε4 carriage	−0.4821	−1.6695	.0983^†^	−1.0701, 0.1375
Age	−0.0143	−1.1410	.2567	−0.0361, 0.0099
Education	0.0118	0.3181	.7511	−0.0530, 0.0803
Total Fruit	0.1926	1.0402	.3009	−0.1465, 0.5432
APOE-ε4 carriage	0.0760	0.4107	.6822	−0.1465, 0.5432
Total Fruit^*^APOE-ε4 carriage	0.0367	0.1134	.9099	−0.5713, 0.6453
Age	−0.0161	−1.2590	.2111	−0.0391, 0.0094
Education	0.0170	0.4463	.6564	−0.0535, 0.0913
FA ratio	0.3197	1.7185	.0889^†^	−0.0225, 0.6676
APOE-ε4 carriage	0.0242	0.1136	.9098	−0.0225, 0.6676
FA ratio^*^APOE-ε4 carriage	−0.0626	−0.2115	.8329	−0.4210, 0.4764
Age	−0.0145	−1.1419	.2563	−0.0360, 0.0101
Education	0.0257	0.6796	.4984	−0.0423, 0.0975
Saturated FA	0.5492	3.0195	.0032^*^	0.2311, 2.1370
APOE-ε4 carriage	0.3644	1.8074	.0738^†^	−0.0803, 0.8192
Saturated FA^*^APOE-ε4 carriage	−0.6638	−2.2520	.0266^*^	−1.2837, −0.0544
Age	−0.0200	−1.6001	.1129	−0.0423, 0.0054
Education	−0.0383	1.0259	.3075	−0.0295, 0.1151
Added sugars	0.1332	0.7107	.4790	−0.1998, 0.4815
APOE-ε4 carriage	0.0999	0.4683	.6406	−0.3593, 0.5589
Added sugars^*^APOE-ε4 carriage	−0.1420	−0.4795	.6327	−0.7403, 0.4467
Age	−0.0165	−1.2816	.2031	−0.0388, 0.0087
Education	0.0175	0.4594	.6470	−0.0488, 0.0874
***Exogenous inhibitory control***				
Total vegetables	0.6932	3.6056	.0005^*^	0.3105, 1.0649
APOE-ε4 carriage	0.3167	1.6152	.1095	−0.0642, 0.6826
Total vegetables^*^APOE-ε4 carriage	−0.8331	−2.7778	.0066^*^	−1.4028, −0.2505
Age	−0.0322	−2.4632	.0155^*^	−0.0557, −0.0052
Education	−0.0699	−1.8123	.0731^†^	−0.1504, 0.0147
Total fruit	−0.1995	−1.0023	.3187	−0.6133, .2133
APOE-ε4 carriage	−0.1899	−0.9541	.3424	−0.5615, 0.1747
Total fruit^*^APOE-ε4 carriage	0.4383	1.2602	.2106	−0.2004, 1.1006
Age	−0.0342	−2.4888	.0145^*^	−0.0588, −0.0057
Education	−0.0649	−1.5842	.1164	−0.1553, 0.0317
FA ratio	0.1914	0.9462	.3464	−0.1928, 0.5889
APOE-ε4 carriage	−0.0875	−0.3780	.7062	−0.5725, 0.4034
FA ratio^*^APOE-ε4 carriage	0.0496	0.1541	.8779	−0.5525, 0.6674
Age	−0.0329	−2.3853	.0190^*^	−0.0574, −0.0038
Education	−0.0565	−1.3722	.1732	−0.1436, 0.0353
Saturated FA	0.3198	1.5866	.1159	−0.0943, 0.7275
APOE-ε4 carriage	0.0659	0.2948	.7688	−0.3653, 0.5231
Saturated FA^*^APOE-ε4 carriage	−0.1880	−0.5754	.5664	−0.8383, 0.4131
Age	−0.0351	−2.5337	.0129^*^	−0.0600, −0.0056
Education	−0.353	−1.2915	.1996	−0.1410, 0.0410
Added sugars	0.5559	2.8741	.0050^*^	0.1653, .9413
APOE-ε4 carriage	0.1546	0.7020	.4844	−0.2826, 0.5778
Added sugars^*^APOE-ε4 carriage	−0.4312	−1.4104	.1617	−0.0156, 0.1515
Age	−0.0360	−2.7048	.0081^*^	−0.0598, −0.0088
Education	−0.0663	−1.6836	.0955^†^	−0.1491, 0.0213
***Endogenous inhibitory control***				
Total vegetables	0.2191	1.0254	.3078	−0.2347, 0.6440
APOE-ε4 carriage	0.2987	1.3706	.1739	−0.1142, 0.7062
Total vegetables^*^APOE-ε4 carriage	−0.5095	−1.5283	.1297	−1.1437, 0.1605
Age	−0.0543	−3.7424	.0003^*^	−0.0809, −0.0208
Education	0.0515	1.2010	.2327	−0.0290, 0.1329
Total fruit	−0.4647	−2.2541	.0265^*^	−0.8666, −0.0466
APOE-ε4 carriage	−0.1719	−0.8338	.4064	−0.6050, 0.2967
Total fruit^*^APOE-ε4 carriage	0.5374	1.4919	.1390	−0.1044, 1.1763
Age	−0.0538	−3.7807	.0003^*^	−0.0806, −0.0219
Education	0.0488	1.1511	.2525	−0.0309, 0.1293
FA ratio	0.3477	1.6398	.1043	−0.0883, 0.7617
APOE-ε4 carriage	0.3039	1.2526	.2134	−0.1786, 0.7866
FA ratio^*^APOE-ε4 carriage	−0.4684	−1.3892	.1680	−1.1087, 0.2002
Age	−0.0538	−3.7253	.0003^*^	−0.0808, −0.0214
Education	0.0655	1.5166	.1326	−0.0155, 0.1443
Saturated FA	0.2034	0.9508	.3441	−0.2200, 0.6625
APOE-ε4 carriage	0.2870	1.2102	.2292	−0.1205, 0.7283
Saturated FA^*^APOE-ε4 carriage	−0.4188	−1.2081	.2300	−1.1130, 0.2592
Age	−0.0565	−3.8507	.0002^*^	−0.0855, −0.0212
Education	0.0653	1.4866	.1404	−0.0222, 0.1565
Added sugars	0.4184	2.0131	.0469^*^	0.0242, 0.8285
APOE-ε4 carriage	0.3713	1.5684	.1201	−0.0968, 0.8777
Added sugars^*^APOE-ε4 carriage	−0.5927	−1.8042	.0743^†^	−1.2302, 0.0149
Age	−0.0558	−3.9006	.0002^*^	−0.0846, −0.0221
Education	0.0488	1.1535	.2516	−0.0326, 0.1285
***Cognitive flexibility***				
Total vegetables	0.1857	0.9197	.3600	−0.2151, 0.5864
APOE-ε4 carriage	0.4843	2.3520	.0207^*^	0.0756, 0.8931
Total vegetables^*^APOE-ε4 carriage	−0.3448	−1.0948	.2763	−0.9700, 0.2804
Age	−0.0274	−1.9980	.0485^*^	−0.0546, −0.0002
Education	−0.0162	−0.4002	.6899	−0.0966, 0.0642
Total Fruit	−0.2748	−1.3972	.1656	−0.6652, 0.1156
APOE-ε4 carriage	0.1702	0.8655	.3889	−0.2201, 0.5605
Total fruit^*^APOE-ε4 carriage	0.3930	1.1438	.2555	−0.2890, 1.0751
Age	−0.0274	−2.0177	.0464^*^	−0.0544, −0.0004
Education	−0.0180	−0.4456	.6569	−0.0984, 0.0623
FA ratio	−0.0093	−0.0458	.9636	−0.4106, 0.3921
APOE-ε4 carriage	0.3280	1.4175	.1596	−0.1313, 0.7873
FA ratio^*^APOE-ε4 carriage	0.0195	0.0607	.9517	−0.6188, 0.6578
Age	−0.0274	−1.9858	.0499^*^	−0.0547, −0.0000
Education	−0.0144	−0.3485	.7283	−0.0961, 0.0674
Saturated FA	0.1874	0.9378	.3507	−0.2092, 0.5840
APOE-ε4 carriage	0.5951	2.6867	.0085^*^	0.1554, 1.0348
Saturated FA^*^APOE-ε4 carriage	−0.5262	−1.6251	.1074	−1.1690, 0.1165
Age	−0.0309	−2.2497	.0268^*^	−0.0581, −0.0036
Education	0.0005	0.0110	.9913	−0.0811, 0.0820
Added sugars	0.4062	2.0783	.0403^*^	0.0182, 0.7942
APOE-ε4 carriage	0.5588	2.5104	.0137^*^	0.1169, 1.0006
Added sugars^*^APOE-ε4 carriage	−0.4669	−1.5115	.1340	−1.0801, 0.1463
Age	−0.0291	−2.1612	.0332^*^	−0.0558, −0.0024
Education	−0.0188	−0.4721	.6379	−0.0978, 0.0602

HEI-2020, Healthy Eating Index-2020; APOE-ε4, apolipoprotein E epsilon 4 allele.

* Indicates statistically significant results (*p* < .05).

† Indicates trends towards significance (*p* < .10).

## Discussion

In this study of middle-aged women with FH+, consistent with hypothesis 1, overall diet quality and select sub-scores were positively associated with overall EF and EF sub-domains. However, contrary to hypothesis 2, overall diet quality and select sub-scores were positively associated with overall EF and EF sub-domains only in APOE-ε4 noncarriers but not in carriers. Specifically, overall HEI-2020 positively predicted overall EF and WM performance in APOE-ε4 noncarriers but did not predict ICex, ICen, or cognitive flexibility in either carriers or noncarriers. Regarding HEI-2020 sub-scores, significant interactions involving APOE-ε4 carriage were found for total vegetables scores and overall EF and ICex, for saturated FA scores and overall EF and WM, and for added sugars scores and ICen. In these cases, better sub-scores (i.e. closer adherence to DGA) were associated with better performance in APOE-ε4 noncarriers with no relationships detected in carriers. These findings suggest that diet quality, a modifiable lifestyle risk factor for AD, and APOE-ε4, a genetic risk factor for AD, may interact to influence EF performance in women at risk of AD during midlife.

The finding of a positive main effect of diet quality is consistent with previous cross-sectional studies. Indeed, in older adults (>60 years, *n* = 3,065) from the 2011–2014 NHANES cohort, diet quality was positively associated with processing speed on the Digit Symbol Substitution Task (DSST),^(9)^ a task similar to one that contributed to (i.e. DSMT) to the computation of ICen composite scores for the present study. In a similar sample (>60 years, *n* = 2,450) from the same NHANES cohort, Fan et al.^(40)^ likewise observed positive associations between diet quality and DSST performance, along with positive effects of whole fruit and seafood and plant protein HEI sub-scores. Notably, Alam et al.^(9)^ reported these effects only among US-born but not foreign-born participants, and Fan et al.^(40)^ found that cognitive benefits of diet quality were limited to non-Hispanic/Latino white participants. Further, in a population-based study of middle-age to older (45–74 years) Hispanic/Latino adults (*n* = 8,461), Estrella et al.^(10)^ observed positive associations between diet quality and verbal memory performance, but not EF (measured via DSST). Taken together, these findings suggest that associations between diet quality and cognition may depend on individual differences (e.g. age, race, ethnicity, genetic risk), that, if unaccounted for, could contribute to variability across studies.

In previous research, diet quality has not been consistently associated with cognitive benefits, which may partially reflect a failure to account for APOE-ε4 carriage. For example, Brouwer-Brolsma et al.^(52)^ examined adults aged 20–70 years (*n* = 1,565) and found that overall diet quality and sub-scores were unexpectedly inversely associated with cognitive performance across memory and EF tasks. Further, Milte et al.^(53)^ did not observe associations between diet quality and measures of EF or memory in middle-aged adults (55–65 years, *n* = 617). Importantly, neither study accounted for APOE-ε4 carriage. Such inverse or null associations may be partially driven by APOE-ε4 carriage, such that positive relationships in APOE-ε4 noncarriers may be present but masked due to null effects in APOE-ε4 carriers, as observed in this study.

Our findings suggest that at midlife, prior to detectable changes in cognitive function, APOE-ε4 carriers may exhibit less sensitivity to the EF benefits associated with better diet quality compared to APOE-ε4 noncarriers. While longitudinal studies are needed to determine the extent to which midlife diet quality influences long-term AD risk in individuals at greater genetic risk, our cross-sectional findings suggest variation in diet quality may differentially affect EF performance in APOE-ε4 noncarriers compared to carriers during midlife. Importantly, EF impairment is commonly observed in early stages of AD and mild cognitive impairment and may predict progression to AD.^(8)^ In a prospective cohort study, Kivipelto et al.^(27)^ observed that APOE-ε4 carriers who were sedentary, consumed low unsaturated FA diets, consumed high saturated FA diets, consumed alcohol frequently, or smoked during midlife had significantly higher odds of developing dementia after an average follow-up of 21 years compared to APOE-ε4 noncarriers who had comparable lifestyle risks. This suggests that the detrimental effects of these lifestyle risk factors on cognitive impairment are amplified by APOE-ε4 carriage. Although the present cross-sectional study does not include AD or dementia outcomes, the observation of differential cognitive associations by APOE-ε4 carriage in a middle-aged sample is particularly concerning, as it suggests that genetically at-risk individuals may not experience the same cognitive benefits from better diet quality during this life stage. Future longitudinal studies initiated during early midlife are necessary to determine whether diet quality influences trajectories of cognitive changes into older age.

The differential effects of APOE-ε4 carriage on EF in the present study may be explained, in part, by increased levels of inflammation^(54,55)^ and disruptions in neuronal and astrocyte lipid metabolism^(56)^ associated with the APOE-ε4 allele. These biological effects of the APOE-ε4 allele have been implicated in accelerating neurodegenerative processes and may influence the extent to which dietary factors influence cognitive outcomes.^(57)^ Although greater intake of flavonoids, anti-inflammatory compounds found in fruits and vegetables,^(58)^ has been implicated in mitigating neurodegenerative disease processes,^(51)^ the presence of the APOE-ε4 allele may result in a higher threshold of diet quality (e.g. consuming high amounts of flavonoid-containing foods) needed to counteract detrimental effects. Similarly, while APOE-ε4 carriers appear responsive to lower saturated and higher polyunsaturated FA intake,^(27)^ disruptions in lipid metabolism may necessitate an even higher threshold of diet quality for cognitive benefits – one that was likely not met in the present study. Indeed, the mean HEI-2020 score in our sample was 66.0 ± 10.7 out of 100, and only nine participants (roughly 8.8%) had HEI-2020 scores above 80, indicating that few participants reported dietary patterns consistent with ‘good’ levels of diet quality (i.e. ≥80).^(3)^ However, a clinically meaningful threshold for HEI-2020 has not been established, and even structured dietary intervention trials typically yield only modest improvements (e.g. four to seven points),^(59)^ suggesting that achieving levels sufficient to offset genetic risk may be particularly challenging.

In parallel with inflammatory and lipid metabolism pathways, diet quality may also influence AD risk through its impact on cardiometabolic health. Specifically, dietary patterns high in saturated FA are associated with heightened cardiovascular disease and type 2 diabetes risk.^(60,61)^ Also, higher vegetable intake is linked to lower obesity and type 2 diabetes prevalence.^(62)^ Recent work also suggests that replacing saturated FA with unsaturated FA sources (e.g. improving saturated FA scores), and decreasing the proportion of energy intake from sugars (e.g. improving added sugars scores) may decrease low-density lipoprotein (LDL) cholesterol levels.^(63)^ Together, these modifiable diet-related dementia risk factors (e.g. LDL cholesterol levels, diabetes, cardiovascular disease, obesity) account for approximately 12% of the population attributable risk during midlife.^(1)^ Thus, promoting diet quality during midlife may be a viable strategy to mitigate dementia risk.

Interestingly, independent of diet quality, APOE-ε4 carriage was associated with better cognitive flexibility. While this contrasts with prior work,^(22,23)^ and detailed discussion regarding the neurobiological mechanisms underlying this finding is outside the scope of the present study, it is consistent with the APOE-ε4 antagonistic pleiotropy hypothesis. This hypothesis posits that APOE-ε4 may impact cognition differently at different life stages such that younger adult carriers may perform better on tasks of cognition than younger adult noncarriers.^(64)^ For example, in a population-level (*n* = 5,561) study including adults aged 45–85 years, Gharbi-Meliani et al.^(65)^ observed that heterozygous (i.e. carrying only one copy of the allele) APOE-ε4 carriers (*n* = 1,412) performed better or equally as well on cognitive tasks compared to noncarriers (*n* = 4,020) up to the age of 70 years (approximately 15 years older than the average age of participants included in the present study), after which, heterozygous APOE-ε4 carriers performed worse than noncarriers.

This study has limitations that should be considered. First, dietary intake was assessed via self-report, a method vulnerable to underreporting consumption of foods perceived as unhealthy.^(66,67)^ However, this challenge may have been partially mitigated by assessing dietary patterns with the HEI-2020 and its sub-scores. For example, in the case of the HEI-2020 derived from food frequency questionnaires, participants are asked how often they consume various food groups and their typical serving sizes over a defined time frame (e.g. past month in the case of the present study). This approach emphasizes habitual dietary intake patterns rather than precise quantities. HEI-2020 scores are also energy-adjusted (e.g. total vegetable intake per 1,000 kcal), helping account for inaccuracies in total energy reporting, providing valid estimates of overall diet quality.^(68)^ Second, because of the reliance on self-report data, overall energy balance could not be reliably accounted for. Given that weight loss, achieved via negative energy balance, supports cardiometabolic health,^(69)^ and poor cardiometabolic health is a key risk factor for AD,^(1)^ diet quality and energy balance may jointly influence neurodegenerative disease risk. Third, the sample included only female and mostly white participants, negating the ability to generalize to males and more diverse populations. Fourth, the cross-sectional design precludes causal inference and limits our ability to draw conclusions related to how the observed associations may change over time. Finally, due to the modest sample size (*n* = 102), statistical power was limited; however, effect sizes for statistically significant interaction terms were small-to-moderate, suggesting the observed effects were meaningful despite limited power. Nevertheless, future replication work is necessary to confirm these findings.

These limitations are balanced by several strengths. First, this study included an at-risk group at a critical time point in the ageing spectrum – middle-aged women with FH+. By characterizing how diet quality, a modifiable risk factor for AD, is associated with EF performance during midlife, a window of time that may precede the accumulation of irreversible neurodegeneration, this work underscores the need to investigate the extent to which dietary interventions should be tailored based on APOE-ε4 carriage. Second, diet quality was assessed using the HEI-2020, a validated, widely accepted measure based on the DGA,^(2)^ making it particularly relevant for a U.S.-based sample. Third, the inclusion of HEI sub-scores enables the examination of the extent to which specific dietary components differentially contribute to cognition, offering a more nuanced understanding than overall scores alone. Finally, by examining APOE-ε4 carriage as a moderator, this study contributes to growing efforts to understand gene-lifestyle interactions, offering insight into how genetic risk influences sensitivity to lifestyle-related risk factors.

While no associations between diet quality and cognitive performance were observed in APOE-ε4 carriers at midlife, it may be the case that a higher threshold of diet quality is necessary for APOE-ε4 carriers to experience cognitive benefits, particularly in middle-aged populations with FH+. Future work using dietary interventions designed to improve diet quality may determine whether significant dietary improvements can positively impact cognition in this population. It is also possible that diet-related cognitive benefits manifest later in life by preventing or delaying cognitive decline in APOE-ε4 carriers, rather than during midlife. Future intervention studies are needed to determine whether dietary improvements can translate to measurable cognitive benefits in genetically at-risk populations. Longitudinal cohort studies have been informative in establishing the relationship between diet, APOE-ε4 carriage, and cognitive decline;^(27,70)^ however, future studies should investigate diet quality indices like HEI-2020 along with its sub-scores and enroll participants during early midlife or younger (e.g. ≤40 years) to effectively characterize how APOE-ε4 and diet quality interact throughout the lifespan. Future work should also study larger, more diverse samples, including males and underrepresented racial/ethnic groups, to enhance generalizability and AD risk stratification across demographic, genetic, and lifestyle factors. Finally, future studies should include AD-relevant memory outcomes (e.g. episodic memory) to more comprehensively explicate the diet quality-cognition relationship.

## Supporting information

10.1017/jns.2026.10106.sm001 Wessinger et al. supplementary material Wessinger et al. supplementary material

## Supplementary material

The supplementary material for this article can be found at https://doi.org/10.1017/jns.2026.10106.

## Table 1. Long description

A table with three columns and eight rows detailing cognitive assessments and their outcomes. The columns are labeled Sub-domain of executive function, Cognitive task, and Outcomes. The sub-domains include Working memory, Exogenous inhibitory control, Endogenous inhibitory control, and Cognitive flexibility. Each sub-domain lists specific cognitive tasks and their corresponding outcomes, such as accuracy scores, reaction times, and total correct responses. The table provides a structured overview of how different cognitive functions are assessed and measured.

Navigate back to Table 1..

## Table 2. Long description

The table compares characteristics of APOE-e4 carriers and noncarriers across various demographic, health, and cognitive metrics. It includes data for 41 carriers and 61 noncarriers, with columns for mean and standard deviation values. Key variables include age, Montreal Cognitive Assessment (MoCA) scores, years of education, body mass index (BMI), race, ethnicity, and several cognitive function scores. Notable trends include similar mean ages (56.7 years for carriers and 57.0 years for noncarriers) and MoCA scores (28.2 for carriers and 28.1 for noncarriers). The table also shows racial distribution, with the majority being White (91.2% for carriers and 91.8% for noncarriers). Cognitive flexibility shows a significant difference with a p-value of 0.024. The table provides a comprehensive overview of the sample characteristics, highlighting the similarities and differences between the two groups.

Navigate back to Table 2..

## Table 3. Long description

The table presents data on the effects of HEI-2020 overall scores and other predictors on composite cognitive scores. It includes five rows and six columns. The columns are labeled Predictor, b, t, p, and CI (LL, UL). The rows detail the predictors for overall executive function, working memory, exogenous inhibitory control, endogenous inhibitory control, and cognitive flexibility. Each row provides values for b, t, p, and confidence intervals (LL, UL) for each predictor. Notable trends include significant effects of HEI-2020 on overall executive function and working memory, with age showing a negative association with executive function and endogenous inhibitory control. Education does not show significant associations with any cognitive scores.

Navigate back to Table 3..

## Table 4. Long description

The table presents detailed results for the HEI-2020 sub-scores, focusing on their effects and those of other predictors on composite cognitive scores. The table includes multiple rows and columns, with each row representing different variables and their respective values. Column headers include categories such as Total, Age, FA score, FA score per 500 grams, etc. Each row provides specific data points for these categories, showing how different factors influence cognitive scores. Notable trends and comparisons are highlighted, such as the impact of age and various health scores on cognitive performance. The table does not include models with BMI as a covariate, as their inclusion did not significantly alter the results.

Navigate back to Table 4..
