# Supplementary material for: APOE-ε4 moderates the association between diet quality and executive function in middle-aged women at increased risk for Alzheimer’s disease
Source: J Nutr Sci. 2026 Jun 8;15:e41. doi: 10.1017/jns.2026.10106 (PMC13244354; doi:10.1017/jns.2026.10106)
Supplement: Wessinger et al. supplementary material [file S2048679026101062sup001.docx]

**Supplemental Material**

| **Supplemental Table 1.** HEI-2020 sub-scores and scoring standards. | | | |
| --- | --- | --- | --- |
| **Component** | **Score Range** | **Maximum Score Standard** | **Minimum Score Standard** |
| Adequacy Sub-Scores | | | |
| Vegetables | 0-5 | ≥ 1.1 cup equivalent per 1000 kcal | No vegetable intake |
| Greens and Beans | 0-5 | ≥ 0.2 cup equivalent per 1000 kcal | No dark green vegetables of legume intake |
| Fruit | 0-5 | ≥ 0.8 cup equivalent per 1000 kcal | No fruit intake |
| Whole Fruit | 0-5 | ≥ 0.4 cup equivalent per 1000 kcal | No whole fruit intake |
| Whole Grains | 0-10 | ≥ 1.5 oz. equivalent per 1000 kcal | No whole grain intake |
| Dairy | 0-10 | ≥ 1.3 cup equivalent per 1000 kcal | No dairy intake |
| Total Protein | 0-5 | ≥ 2.5 oz equivalent per 1000 kcal | No protein foods intake |
| Seafood and Plant Protein | 0-5 | ≥ 0.8 oz equivalent per 1000 kcal | No seafood or plant protein foods intake |
| FA Ratio | 0-10 | (PUFA + MUFA) / SFA ≥ 2.5 | (PUFA + MUFA) / SFA ≤ 1.2 |
| Discretionary Sub-Scores | | | |
| Sodium | 0-10 | ≤ 1.8 oz equivalent per 1000 kcal | ≥ 4.3 oz equivalent per 1000 kcal |
| Refined Grains | 0-10 | ≤ 1.1 g per 1000 kcal | ≥ 2.0 g per 1000 kcal |
| Saturated FA | 0-10 | < 6.5% of total kcal intake | ≥ 26% of total kcal intake |
| Added Sugar | 0-10 | ≤ 8.0% of total kcal intake | ≥ 16% of total kcal intake |
| HEI-2020, Healthy Eating Index-2020; FA, fatty acids; PUFA, polyunsaturated fatty acids; MUFA, monounsaturated fatty acids.  Fruit includes 100% fruit juice; Whole Fruit includes all forms excluding juice; Greens and Beans includes beans, peas, and lentils; Dairy includes all milk products (e.g., milk, yogurt, cheese, fortified soy beverages); Seafood and Plant Protein includes seafood, nuts, seeds, soy, beans, peas, and lentils. Adapted from USDA: Food and Nutrition Service, (2023): How the HEI is Scored. | | | |


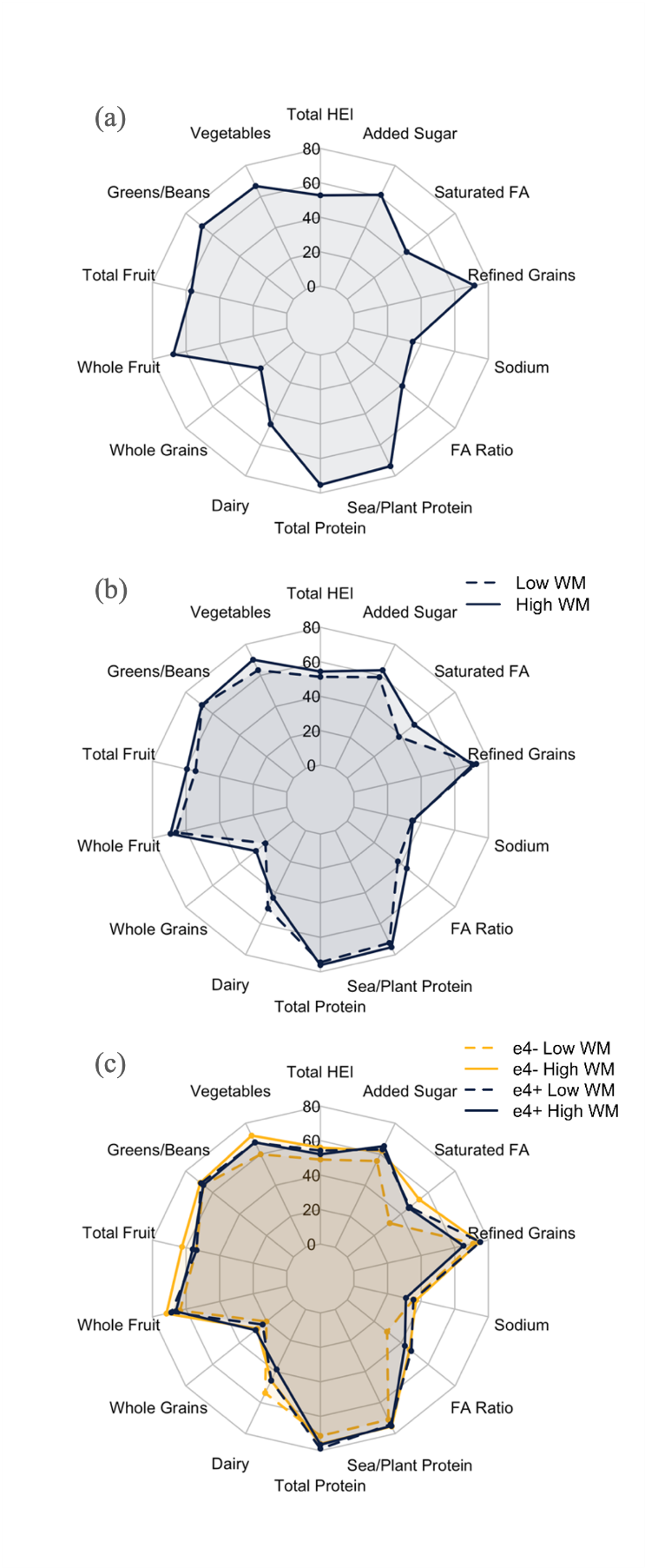

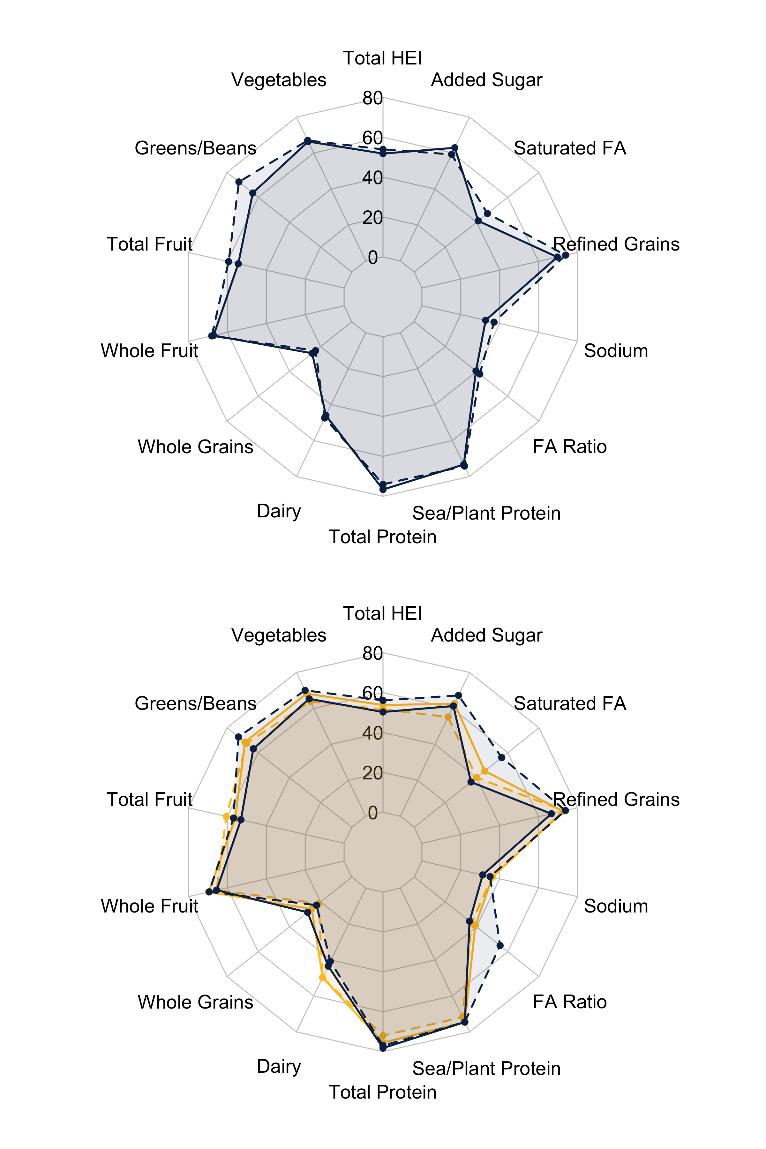

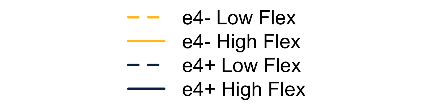

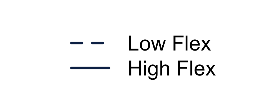


(a)

(b)

(c)

(d)

**Supplemental Figure 1.** HEI-2020 radar plots across (a) low vs high working memory performers, (b) low vs high working memory performers stratified by APOE-ε4 carrier status, (c) low vs high cognitive flexibility performers, and (d) low vs high cognitive flexibility performers stratified by APOE-ε4 carrier status.

e4-, APOE-ε4 noncarriers; e4+, APOE-ε4 carriers; WM, composite working memory scores; Flex, composite cognitive flexibility scores; FA, fatty acids; HEI, Healthy Eating Index-2020.

**
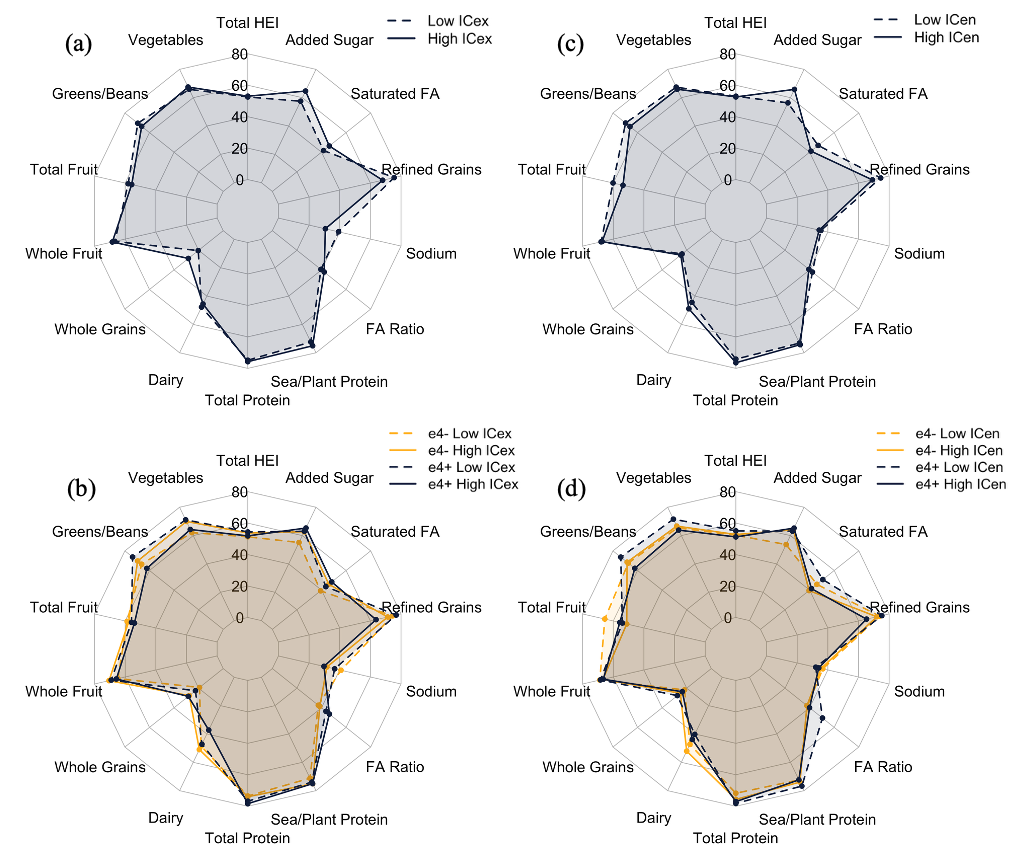
**

**Supplemental Figure 2.** HEI-2020 radar plots across (a) low vs high exogenous inhibitory control performers, (b) low vs high exogenous inhibitory control performers stratified by APOE-ε4 carrier status, (c) low vs high endogenous inhibitory control performers, and (d) low vs high endogenous inhibitory control performers stratified by APOE-ε4 carrier status.

e4-, APOE-ε4 noncarriers; e4+, APOE-ε4 carriers; ICen, composite endogenous inhibitory control scores; ICex, composite exogenous inhibitory control scores; FA, fatty acids; HEI, Healthy Eating Index-2020.

| Supplemental Table 2. HEI-2020 sub-scores stratified by APOE-ε4 carrier status. | | | | | |
| --- | --- | --- | --- | --- | --- |
| **HEI-2020 Sub-Score** | **APOE-ε4 Carriers (n = 41)** | | **APOE-ε4 Noncarriers (n = 61)** | | **Significance** |
|  | **Mean** | **SD** | **Mean** | **SD** |  |
| *Adequacy HEI-2020 Components* | | | | | |
| Vegetables | 4.2 | 0.9 | 4.1 | 1.0 | .623 |
| Greens and Beans | 4.2 | 1.3 | 4.3 | 1.3 | .953 |
| Fruit | 3.4 | 1.5 | 3.6 | 1.6 | .501 |
| Whole Fruit | 4.2 | 1.4 | 4.2 | 1.3 | .947 |
| Whole Grain | 3.2 | 2.1 | 2.9 | 2.2 | .559 |
| Dairy | 5.3 | 2.3 | 6.2 | 2.4 | .046* |
| Total Protein | 4.9 | 0.5 | 4.6 | 0.8 | .062^†^ |
| Seafood and Plant Protein | 4.7 | 0.9 | 4.6 | 0.9 | .664 |
| FA Ratio | 5.6 | 2.8 | 4.8 | 3.3 | .151 |
| *Moderation HEI-2020 Components* | | | | | |
| Sodium | 4.1 | 2.5 | 4.5 | 3.0 | .494 |
| Refined Grains | 8.8 | 1.8 | 9.1 | 1.7 | .340 |
| Saturated FA | 5.8 | 3.5 | 5.3 | 3.3 | .550 |
| Added Sugars | 8.0 | 2.6 | 7.4 | 2.9 | .240 |
| HEI-2020, Healthy Eating Index-2020; APOE-ε4, apolipoprotein E epsilon 4 allele; FA, fatty acids.  * Indicates statistically significant results (*p* < .05).  ^†^ Indicates trend towards significance (*p* < .10). | | | | | |


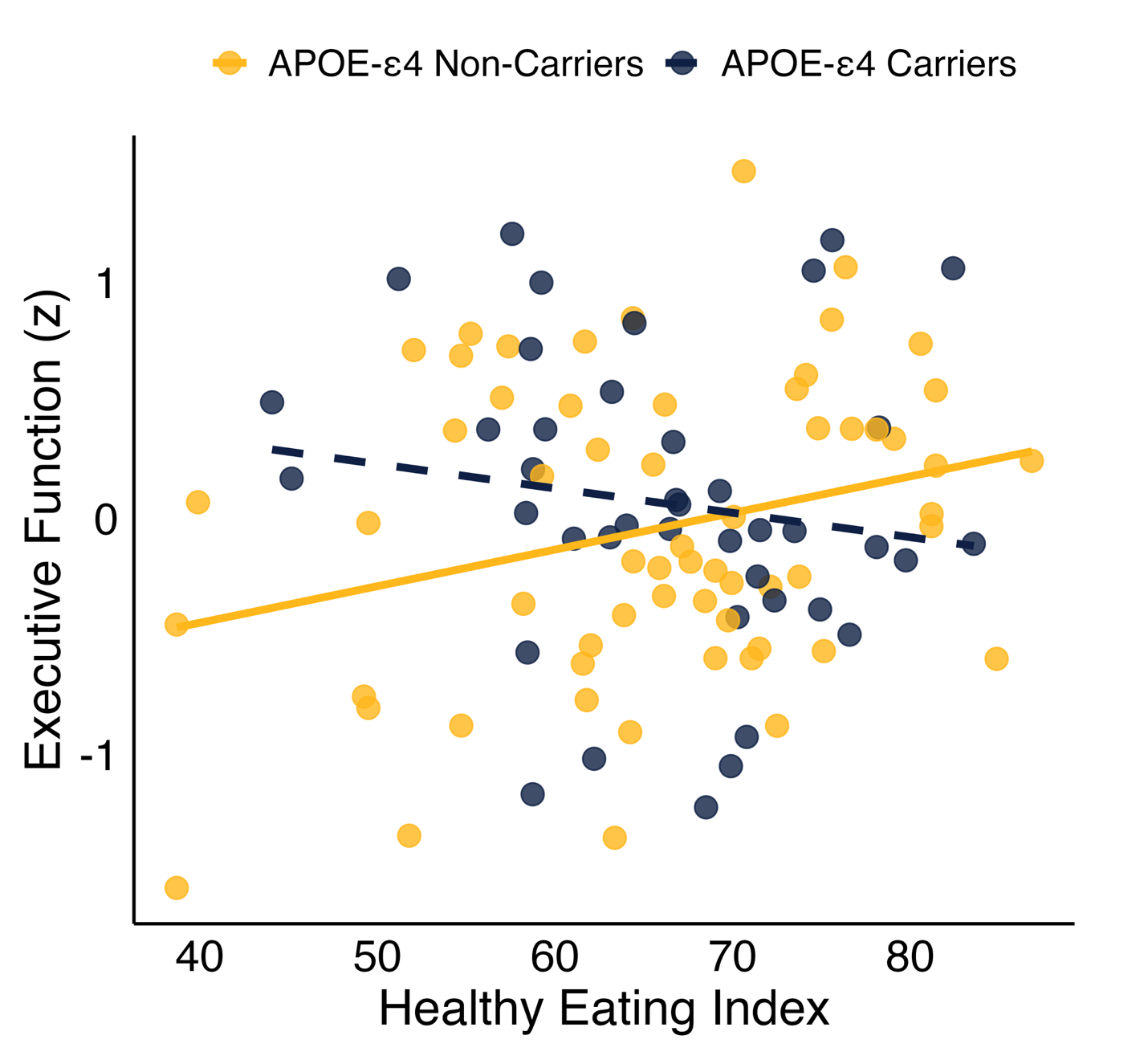


**Supplemental Figure 3**. Scatter plot with fitted regression lines stratified by APOE-ε4 carrier status illustrating the association between HEI-2020 and overall executive function. while simple slopes derived from the moderation analyses are presented in Figure 3a-b, this figure displays raw, individual participant data with unadjusted regression lines.


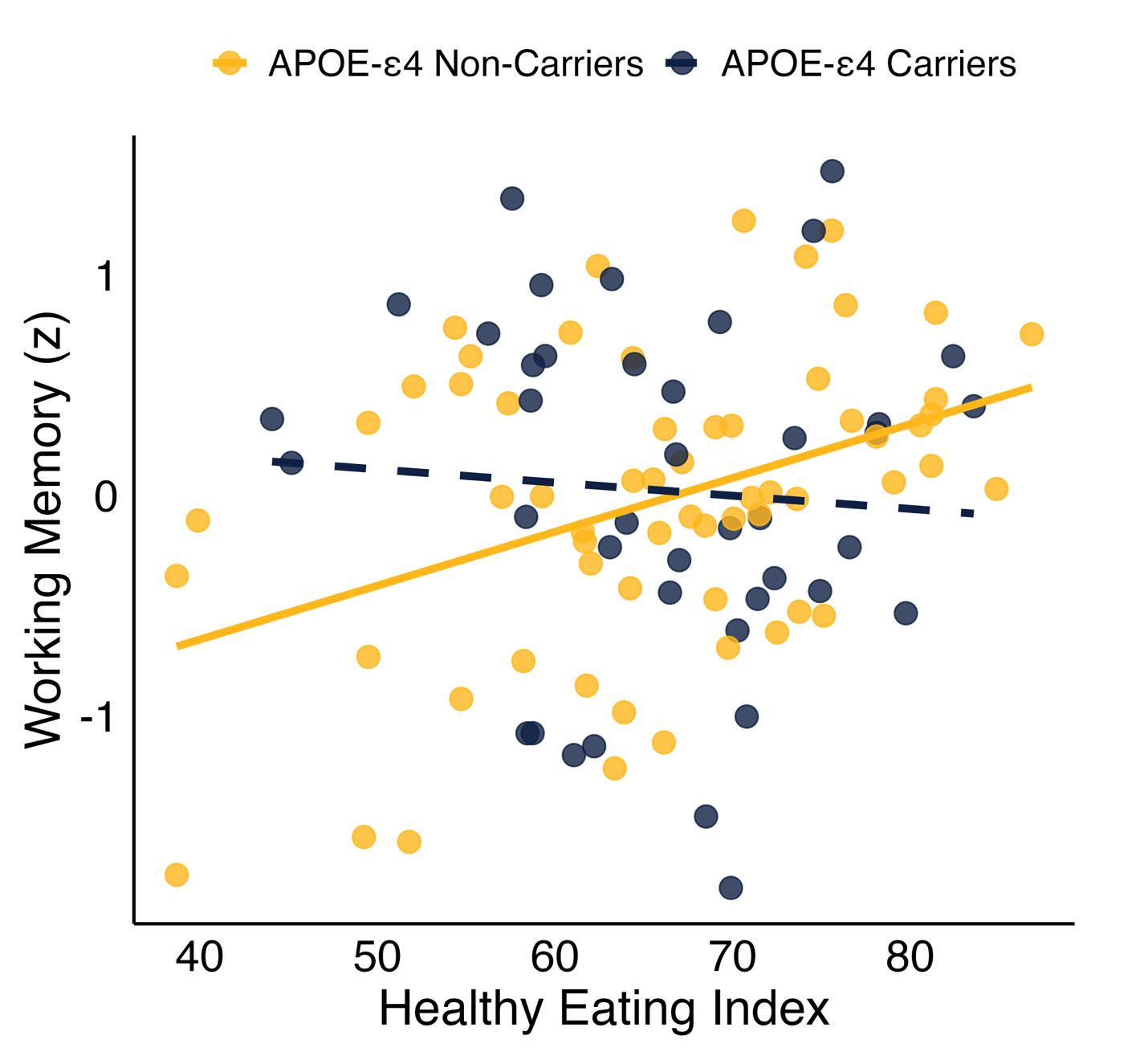


**Supplemental Figure 4**. Scatter plot with fitted regression lines stratified by APOE-ε4 carrier status illustrating the association between HEI-2020 and working memory. while simple slopes derived from the moderation analyses are presented in Figure 3a-b, this figure displays raw, individual participant data with unadjusted regression lines.

| **Supplemental Table 3.** Conditional effects of overall HEI-2020 scores and sub-scores on composite cognitive performance scores stratified by APOE-ε4 carrier status. | | | | | |
| --- | --- | --- | --- | --- | --- |
| **APOE-ε4 Carriage** | ***b*** | ***SE*** | ***t*** | ***p*** | ***CI (LL, UL)*** |
| ***Overall Executive Function*** | | | | | |
| *Overall HEI-2020* | | | | | |
| Noncarriers | 0.0242 | 0.0081 | 3.0016 | 0.0034* | 0.0082, 0.0403 |
| Carriers | -0.0066 | 0.0122 | -0.5378 | 0.5920 | -0.0309, 0.0177 |
| *Vegetables* | | | | | |
| Noncarriers | 0.4091 | 0.1584 | 2.5821 | 0.0113* | 0.0946, 0.7236 |
| Carriers | -0.1292 | 0.1912 | -0.6758 | 0.5008 | -0.5086, 0.2503 |
| *Saturated FA* | | | | | |
| Noncarriers | 0.3640 | 0.1592 | 2.2869 | 0.0244* | 0.0481, 0.6799 |
| Carriers | -0.1346 | 0.1959 | -0.6871 | 0.4937 | -0.5234, 0.2542 |
| ***Working Memory*** | | | | | |
| *Overall HEI-2020* | | | | | |
| Noncarriers | 0.0223 | 0.0077 | 2.8876 | .0048* | 0.0070, 0.0377 |
| Carriers | -0.0066 | 0.0123 | -0.5377 | .5920 | -0.0310, 0.0178 |
| *Saturated FA* | | | | | |
| Noncarriers | 0.5492 | 0.1819 | 3.0195 | .0032* | 0.1882, 0.9102 |
| Carriers | -0.1146 | 0.2239 | -0.5119 | .6099 | -0.5590, 0.3298 |
| ***Exogenous Inhibitory Control*** | | | | | |
| *Vegetables* |  |  |  |  |  |
| Noncarriers | 0.6932 | 0.1923 | 3.6056 | .0005* | 0.3116, 1.0748 |
| Carriers | -0.1399 | 0.2319 | -0.6033 | .5477 | -0.6003, 0.3205 |
| ***Endogenous Inhibitory Control*** | | | | | |
| *Added Sugar* | | | | | |
| Noncarriers | 0.4184 | 0.2079 | 2.0131 | .0469* | 0.0058, 0.8310 |
| Carriers | -0.1743 | 0.2541 | -0.6859 | .4944 | -0.6787, 0.3301 |
| APOE-ε4, apolipoprotein E epsilon 4 allele; CI, confidence interval; HEI-2020, Healthy Eating Index-2020; FA, fatty acids.  * Indicates statistically significant results (*p* < .05). | | | | | |

| **Supplemental Table 4**. Correlation matrix. | | | |
| --- | --- | --- | --- |
|  | BMI | HEI-2020 | Education |
| BMI |  |  |  |
| HEI-2020 | -0.059 |  |  |
| Education | 0.008 | 0.122 |  |

| **Supplemental Table 5**. p-value matrix. | | | |
| --- | --- | --- | --- |
|  | BMI | HEI-2020 | Education |
| BMI |  |  |  |
| HEI-2020 | .556 |  |  |
| Education | .933 | .224 |  |

| **Supplemental Table 6.** Effects of HEI-2020 overall score and the other predictors on overall EF composite score. | | | | |
| --- | --- | --- | --- | --- |
| **Predictor** | ***b*** | ***t*** | ***p*** | ***CI (LL, UL)*** |
| ***Overall Executive Function***  *R^2^* = .135, *F* (6, 95) = 2.475, *p* = .029* | | | | |
| HEI-2020 | 0.0149 | 2.0914 | 0.0392* | 0.0008, 0.0290 |
| APOE-ε4 carriage | 0.1003 | 0.8084 | 0.4209 | -0.1460, 0.3465 |
| HEI-2020*APOE-ε4 carriage | -0.0243 | -1.8920 | 0.0615^†^ | -0.0498, 0.0012 |
| Age | -0.0278 | -2.5500 | 0.0124* | -0.0495, -0.0062 |
| Education | 0.0078 | 0.2376 | 0.8127 | -0.0571, 0.0727 |
| BMI | -0.0089 | -0.7742 | 0.4407 | -0.0319, 0.0140 |
| CI=confidence interval; HEI-2020, Healthy Eating Index-2020; APOE-ε4, apolipoprotein E epsilon 4 allele.  * Indicates statistically significant results (*p* < .05).  ^†^ Indicates trend towards significance (*p* < .10). | | | | |
